# Highly monodisperse Pd-Ni nanoparticles supported on rGO as a rapid, sensitive, reusable and selective enzyme-free glucose sensor

**DOI:** 10.1038/s41598-019-55746-y

**Published:** 2019-12-17

**Authors:** Aysun Şavk, Kemal Cellat, Kubilay Arıkan, Fatih Tezcan, Senem Karahan Gülbay, Servet Kızıldağ, Elif Şahin Işgın, Fatih Şen

**Affiliations:** 1 Sen Research Group, Biochemistry Department, Faculty of Arts and Science, Dumlupınar University, Evliya Çelebi Campus, 43100 Kütahya, Turkey; 2 Mersin University, Science and Letters Faculty, Chemistry Department, 33343 Mersin, Turkey; 3 Department of Chemistry, Faculty of Sciences, Dokuz Eylul University, Buca İzmir, Turkey; 4 College of Vocational School of Health Services, Dokuz Eylül University School of Medicine, İzmir, Turkey

**Keywords:** Electronic properties and devices, Electrocatalysis

## Abstract

In this work, highly monodispersed palladium-nickel (Pd-Ni) nanoparticles supported on reduced graphene oxide (rGO) were synthesized by the microwave-assisted methodology. The synthesized nanoparticles were used for modification of a glassy carbon electrode (GCE) to produce our final product as PdNi@rGO/GCE, which were utilized for non-enzymatic detecting of glucose. In the present study, electrochemical impedance spectroscopy (EIS), chronoamperometry (CA) and, cyclic voltammetry (CV) methods were implemented to investigate the sensing performance of the developed glucose electrode. The modified electrode, PdNi@rGO/GCE, exhibited very noticeable results with a linear working range of 0.05–1.1 mM. Moreover, an ultralow detection limit of 0.15 μM was achieved. According to the results of amperometric signals of the electrodes, no significant change was observed, even after 250 h of operation period. In addition, the highly monodisperse PdNi@rGO/GCE was utilized to electrochemical detection of glucose in real serum samples. In light of the results, PdNi@rGO/GCE has shown an excellent sensing performance and can be used successfully in serum samples for glucose detection and it is suitable for practical and clinical applications.

## Introduction

In today’s world, there are many chronic diseases that people struggling, and diabetes is one of them. High levels of blood sugar can be a sign of diabetes which considered one of the most important health problems worldwide. Since 1960, very important developments have been progressed in the determination of biomolecules through electrochemical systems^1–3^. Those electrochemical systems were applied in a wide range of applications, for instance, glucose oxidation, therapeutic approaches, water treatment, biofuel cell development and other analytical approaches in the food sector^4–8^. Glucose oxidation is considered a major step in the oxidation of organic materials and has been studied by many scientists^9–11^.

The development of novel materials that can be used in electrocatalytic processes still has great importance^12,13^. In order to achieve electrochemical detection of glucose, enzymatic glucose sensors are commonly used^14–17^. However, enzymatic glucose sensors have some disadvantages, for instance, being expensive, discontinuity, and immobilization problem. For example, glucose oxidase, one of the most commonly used enzyme for enzymatic glucose detection, it rapidly loses its activity and can be permanently damaged at higher operation temperatures (> 40 °C) if pH is less than 2 or higher than 8. Moreover, humidity can affect these enzymatic sensors. Incorrectly measured levels of glucose might cause prescribing overdose of insulin which leads hypoglycemia^18^. The stability problems in enzymatic systems hinder the applicability and development of these systems^19^. Non-enzymatic glucose sensors are better alternatives due to their superior properties, such as cost-effectiveness, high sensitivity, better performance in glucose oxidation, and stability. In literature, there are various nanotechnology applications developed for the detection of glucose; metals (Au, Ni, Co, Cu, In, or Ru), metal oxides (cobalt (II) oxide, nickel oxide hydroxide, or ruthenium (IV) oxide), and metallic composites (Au-Pt, Cr-Ni, Cu-Pd)^12,20–23^ are among them. In addition, several carbon-based compounds, such as carbon nanotubes (CNTs) and graphene, have been proposed to utilized as electrocatalysts for oxidation of glucose and increase the reaction yield on the surface of the sensor^3,24,25^. Among these carbon-based support materials, reduced graphene oxide (rGO) has unique properties such as good electrical conductivity, wide surface area, mechanical and chemical stability. These properties enable the rGO or rGO-containing composites, favorable for sensor applications. rGO is a very suitable supporting material for biosensors and electrochemical sensors^26–28^. Recently, metal nanoparticles and rGO composites have been presented, and they exhibited high sensitivity in sensing small molecules, such as H_2_O_2_, glucose, and methanol. A very few studies reported the using of rGO as a catalyst with very high sensitivity, selectivity, and durability. However, there is no study on the using of PdNi-rGO combination as a glucose sensor^29–31^. We also tested the stability of synthesized PdNi@rGO due to Ostwald ripening effect which may cause small metal particles can be detached from carbonous support and clustered to form larger particles^32^.

This study aims to the utilization of reduced graphene oxide with PdNi nanoparticles (PdNi@rGO) to enhance the corresponding activity of glucose detection. For this purpose, highly monodisperse PdNi@rGO was used to modify a glassy carbon electrode (GCE). PdNi@rGO/GCE was developed for the detection of glucose in biologic samples as a non-enzymatic sensor. Fabrication and characterization of the developed sensor were demonstrated by high resolution-transmission electroscope microscopy (HR-TEM), Raman spectroscopy, X-ray diffraction (XRD), and X-ray photoelectron spectroscopy (XPS). Prepared nanoparticle enhanced electrode was examined by electrochemical techniques to uncover its selectivity, stability, sensitivity and detection limit as a non-enzymatic biosensor for glucose detection.

## Experimental

### Synthesis of highly monodisperse PdNi@rGO nanoparticles

A modified microwave synthesis method adapted from previous studies^33–36^ in literature was conducted for a consistent producing of monodisperse nanomaterials. For this aim, equal amounts (0.25 mmol of each) of PdCl_2_ and Ni(Ac)_2_ were dissolved in 20 mL ethylene glycol with vigorous mixing, in the presence of oleylamine (OA) which is used for stabilization of metal nanoparticles. The pH adjustment was achieved using a NaOH-ethylene glycol solution. In this work, ethylene glycol was added for reducing of PdCl_2_ and Ni(Ac)_2_. The mixture was located in a microwave oven for 60 s (5 times) at 1200 W. Finally, the resultant was filtered and washed using deionized water and acetone. Synthesis of GO and rGO is given in the Supporting Information section. The produced PdNi bimetallic nanoparticles were mixed with (0.50 mmol) rGO using a sonicator to produce a uniform dispersion. The solution was then subjected to microwave irradiation (1200 W) for another 60 s (5 times). Obtained PdNi@rGO nanoparticles were dried under vacuum at room temperature.

### Preparation of PdNi@rGO/GCE modified electrode

The GC electrode surface was polished to mirror-like using 0.3-µm and 0.05-µm Al_2_O_3_ powder and washed with distilled H_2_O and ethanol for 2 min. 1 mg PdNi@rGO were dispersed in 10 mL N,N-dimethylformamide (DMF) in an ultrasonic bath, and a black solution obtained. 20 µL of the solution was cast at the surface of the glassy carbon electrode and the solvent was evaporated at 50 °C.

## Results and Discussion

### Characterization of nanoparticles

**Figure 1:**
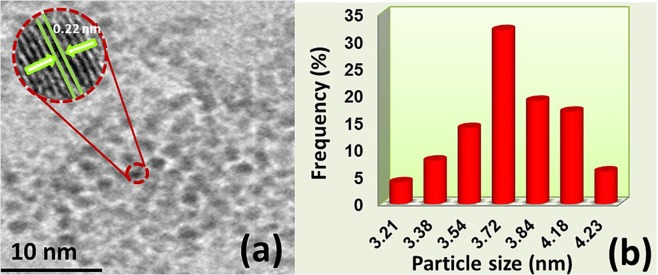
(**a**) TEM and HR-TEM image (inlay); (**b**) particle size histogram for PdNi@rGO.

HR-TEM, Raman, XRD, and XPS analyses were utilized to characterize PdNi@rGO nanocomposites, the detailed examination of characterization methods was given in Supporting Information section (see Appendix). TEM and HR-TEM images of PdNi@rGO was given in Fig. 1(a). As can be seen, PdNi nanoparticles were monodispersely distributed on the surface of rGO and no agglomeration was observed. The synthesized nanoparticles were mostly spherical shaped and have an average particle size of 3.72 ± 0.51 nm (Fig. 1(b)). In addition, HR-TEM analysis presented the atomic lattice fringe of 0.22 nm and this is totally in agreement with the nominal Pd (111) range^37,38^.

**Figure 2:**
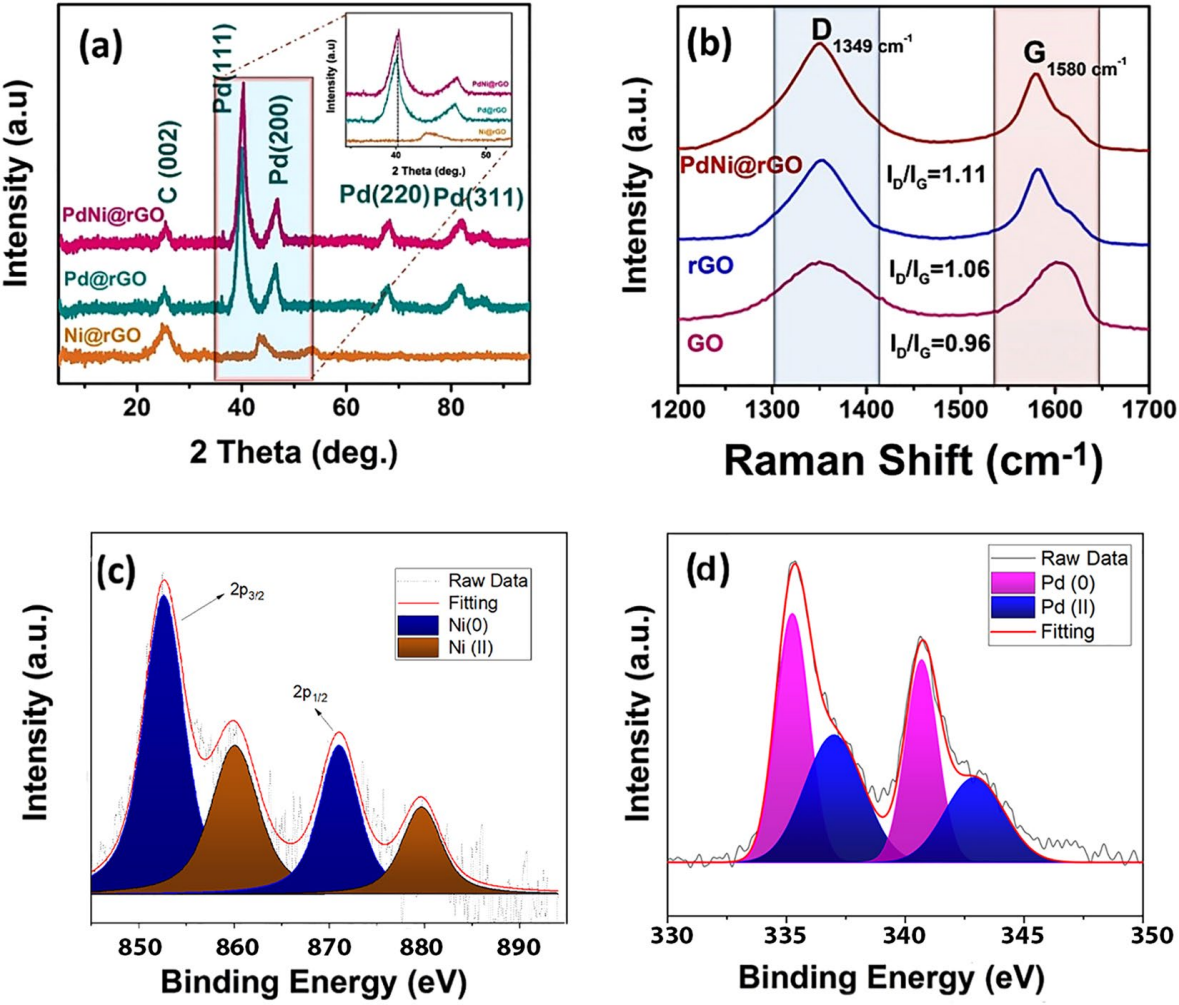
(**a**) The XRD and (**b**) Raman spectra of PdNi@rGO NPs; XPS spectra of (**c**) Ni 2p and (**d**) Pd 3d.

XRD analyses were implemented to identify the structure of crystal formations and the average crystal size of PdNi@rGO. In Fig. 2(a), typical crystal structures observed in our samples can be seen. The crystal planes of Pd (111), (200), (220) and (311) are attributed to 2θ peaks of 40.3°, 46.4°, 68.4°, and 82.5°, respectively, which reveals a face-centered cubic (fcc) crystal structure. The diffraction peaks in Fig. 2(a) exhibited higher 2θ values (slightly shifted when compared with the pure Pd), this indicates the existence of the monodisperse PdNi@rGO alloy. Additionally, the peak observed at 25.5° implying that the reduction of GO to reduced graphene oxide was achieved successfully. The XRD results of PdNi revealed that there was no significant nickel diffraction peak, due to the fact that synthesized nanoparticle has stronger signals for the palladium, which is associated with the amorphous structure. Using the Eq. 1, the average size of crystal particles was calculated as 3.21 ± 0.51 nm. The results are compatible with the result obtained from TEM analysis and also previous studies in the literature^39–43^.

1 $${\rm{d}}({\mathbb{\mathring{\rm A} }})=\frac{k\lambda }{\beta \,\cos \,\theta }$$

Where k is the coefficient (0.9), $\lambda$ is the wavelength of the X-ray (= 1.54056 Ǻ), β is the full width of the respective diffraction peak at the half-maximum (rad), and θ is the angle at the position of peak maximum (rad). With the aim of finding the lattice parameter values (αPdNi), the diffraction peak of PdNi (220) was used. By the using of Eq. 2, the lattice parameter value of the PdNi@rGO was found to be 3.88 Å, which is a little lower than 3.89 Å (for pure Pd)^44,45^.

2 $$\mathrm{Sin}\,{\theta }=\frac{{\boldsymbol{\lambda }}\sqrt{{{\boldsymbol{h}}}^{2}+{{\boldsymbol{k}}}^{2}+{{\boldsymbol{l}}}^{2}}}{2{\boldsymbol{a}}}\,({\rm{for}}\,{\rm{cubic}}\,{\rm{structure}})$$

Raman analysis conducted for distinguishing irregular and regular carbon structures. Raman spectra of GO, rGO, and PdNi@rGO were displayed in Fig. 2(b). Two distinct peaks observed at 1349 cm^−1^ and 1580 cm^−1^ corresponding to D (E_2g_ phonon) and G (A_1g_ symmetry) bands. The D band is related to the disordered carbon atoms, while the G band is associated to sp2-hybridized graphitic carbon atoms. The I_D_/I_G_ ratios (the intensity ratio of the D band to the G band which is used for seeing modifications and defects in nanomaterials) were calculated as 0.96, 1.06 and 1.1 for GO, rGO and PdNi@rGO, respectively. The increasing I_D_/I_G_ ratio indicates that GO deoxygenated, and rGO became functionalized with PdNi nanoparticles.

XPS analysis was utilized to investigate the surface composition and the oxidation states of Pd and Ni atoms in PdNi@rGO. The XPS spectra for Pd 3d and Ni 2p were displayed in Fig. 2(c,d). The Gaussian-Lorentzian method was employed to examine the Pd 3d and Ni 2p regions of the spectrum. The relative density of the species was assessed by counting the integral of peaks. The binding energies (±0.3 eV) were measured by reference to the C 1 s peak at 283.6 eV. The peaks observed at 852.6 eV/871.0 eV and 860.0 eV/879.7 eV corresponded to metallic Ni and Ni (II), respectively (Fig. 2(c)). Similarly, in Fig. 2(d), peaks observed at 335.3 eV/340.7 eV and 337.0 eV/342.9 eV corresponded to metallic Pd and Pd (II), respectively. The intensity of the peaks showed that Pd and Ni exist predominantly in metallic form. The peaks of Pd(II) and Ni(II) observed in XPS spectra are the probable results of chemical sorption of oxygen or surface oxidation while nanomaterials were producing. A low energy shift of Ni 2p_3/2_ peak indicates an alloying process of PdNi.

### Electrochemical properties of the PdNi@rGO/GCE

#### Electrochemical impedance characterization of electrodes

**Figure 3:**
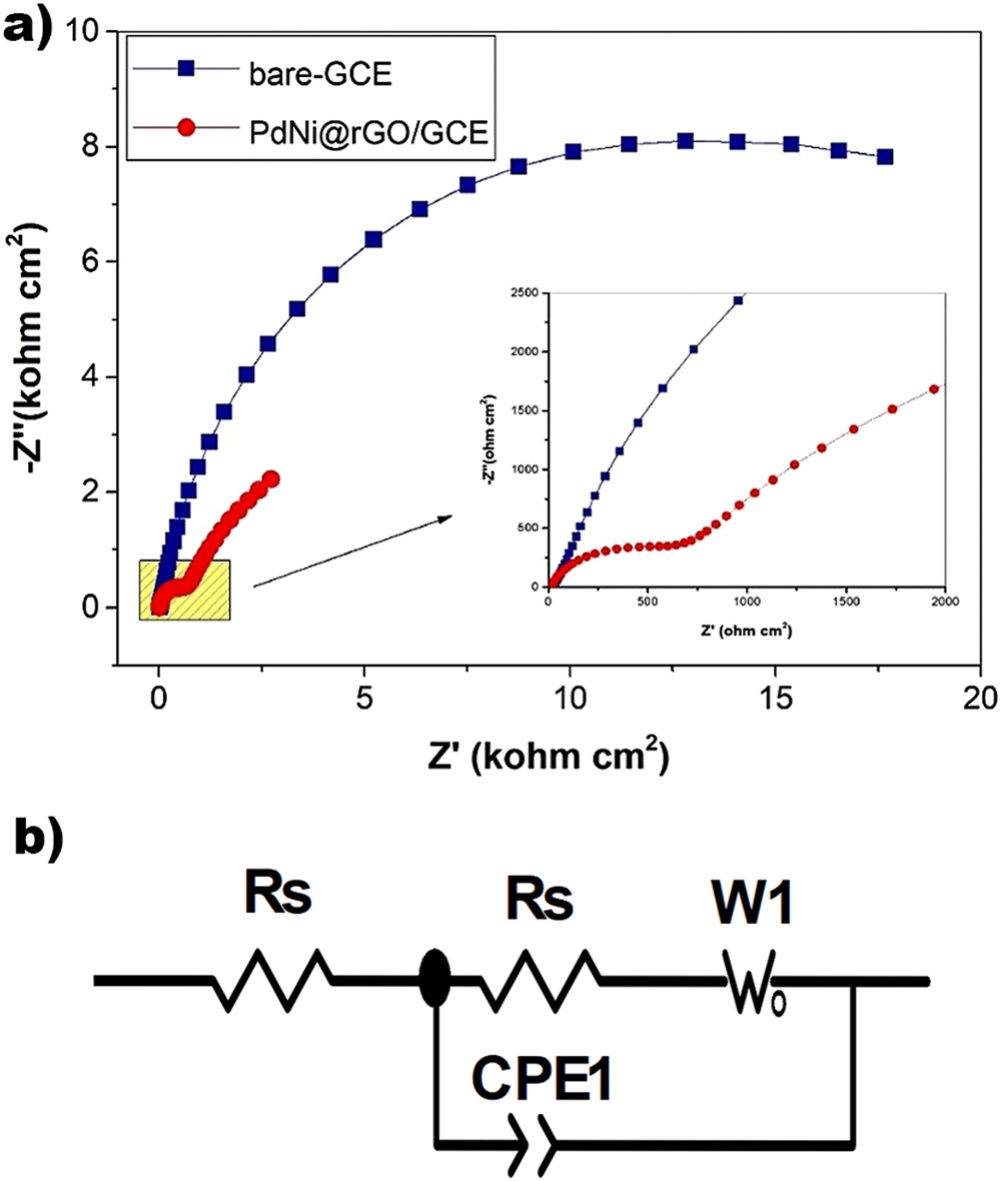
(**a**) Nyquist plots of bare-GCE and Pd-Ni@rGO modified electrodes, (**b**) equivalent circuit diagram.

The bare-GCE and Pd-Ni@rGO modified electrodes were tested with EIS. Nyquist plots of the bare and modified electrodes were shown in Fig. 3(a), and related electrical equivalent circuit diagram was shown in Fig. 3(b). The EIS data were fitted by Zview software. According to fitted EIS results, Pd-Ni@rGO modified electrodes exhibited a nearly straight line with significantly lower charge-transfer resistance (Rct = 600 Ω cm^2^), while the bare-GCE exhibited a semi-circle in the high-frequency range (Rct = 19844 Ω cm^2^). This indicates the prepared Pd-Ni@rGO enhanced electrochemical process on the electrode/electrolyte interface in contrast to a relatively slow electrochemical performance of bare GCE. Our findings support that electrical conductivity and electron transfer rates are increased as a result of decreasing charge transfer resistance.

#### Cyclic voltammetry measurement results

**Figure 4:**
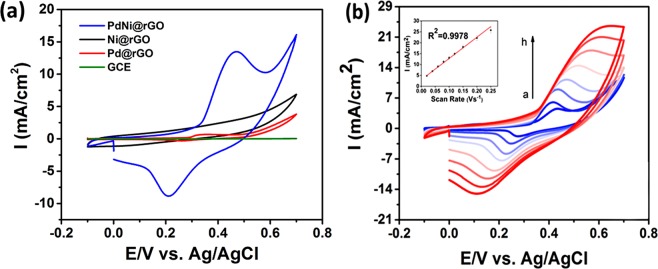
(**a**) Cyclic voltammograms of GCE, Pd@rGO, Ni@rGO and PdNi@rGO (scan rate: 50 mV s^−1^, in 0.1 M NaOH and 0.1 mM glucose). (**b**) Cyclic voltammograms of PdNi@rGO/GCE at different scanning rates. (1 mM glucose, and scan rates from 20 to 180 mV s^−1^).

In order to analyze the glucose oxidation in bare and modified glassy carbon electrodes(GCE), cyclic voltammetry (CV) measurements were conducted. Figure 4(a) represents the comparative results of CV experiments. Electrochemical activities of GCE, monometallic electrodes (Ni@rGO/GCE and Pd@rGO/GCE), and PdNi@rGO/GCE electrode were measured at the coexistence of 0.1 mM glucose in 0.1 M NaOH medium. All the CV tests were conducted in a potential range of −0.2 V to + 0.8 V and at 50 mV s^−1^ scan rate. For Ni@rGO/GCE and Pd@rGO/GCE modified electrodes, intensities of the anodic and cathodic peaks were found to be close. However, for the PdNi@rGO/GCE modified electrode, the intensity of the cathodic and anodic peaks exhibited a significant increase. This can be explained by the larger surface area of PdNi@rGO/GCE and enhanced electron transfer rate. Moreover, the anodic peak current of the modified PdNi@rGO/GCE electrode was higher than the PdNi/GCE electrode (Fig. S2). The results demonstrated that the PdNi@rGO/GCE showed enhanced electrochemical activity for glucose electrooxidation to gluconolactone compared to GCE and modified with monometallic nanoparticles.

The electrochemical properties of PdNi@rGO/GCE were also examined at various scan rates using 0.3 mM glucose in 0.1 M NaOH (Fig. 4(b)). An increased redox current was achieved by increasing scan rate from 20 to 180 mV s^−1^. This is explained by a diffusion-controlled redox reaction that occurred on PdNi@rGO/GCE. Here, it should be noted that 0.1 M NaOH is used for all electrochemical experiments and it can be expected a possible shift to positive potential values.

**Figure 5:**
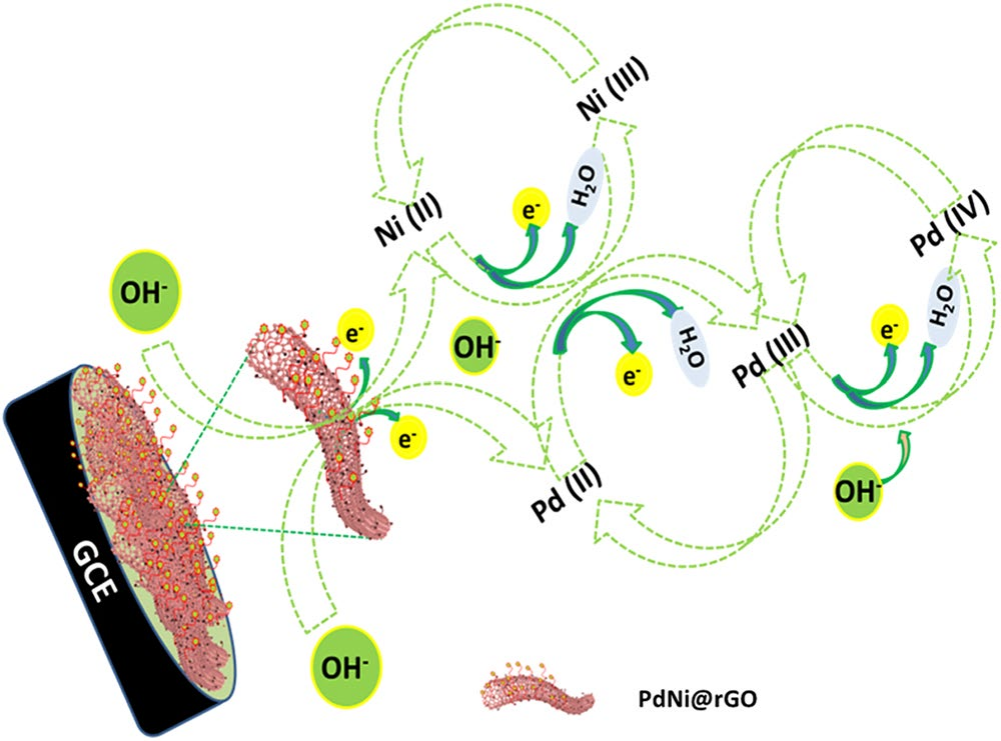
The mechanism for the activation of Ni-Pd@rGO/GCE in the alkali medium.

The mechanism for the activation of PdNi@rGO/GCE was presented in Fig. 5: (i) anodic scan (under the alkaline conditions), Pd (III) and Ni (III) were formed from the Pd (II) and Ni (II); (ii) at higher potentials, Pd (IV) was formed from Pd (III); and (iii) in the cathodic scan, Pd (II) and Ni (II) formation was observed.

#### Amperometric measurements and optimization studies

**Figure 6:**
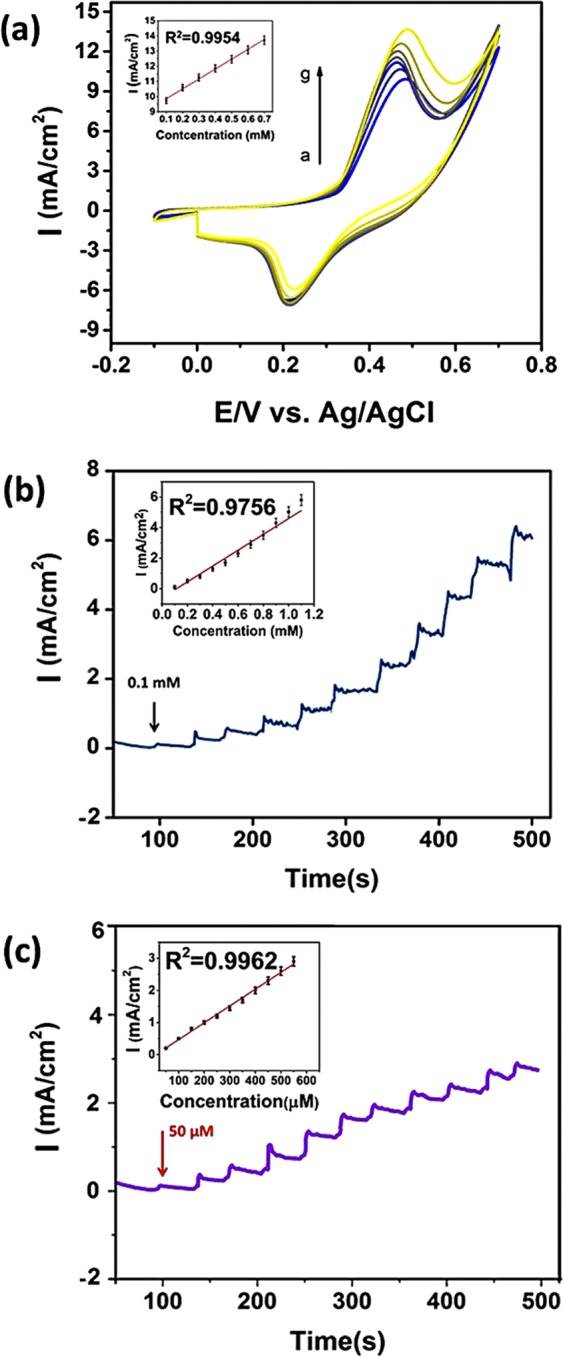
(**a**) CVs for the PdNi@rGO/GCE at various concentrations of glucose (0.1–0.7 mM). The linear range plot was shown in the inset. (**b**) Amperometric i-t curve responses (0.1 to 1.1 mM), inset shows the corresponding calibration plots (**c**) Amperometric i-t curve responses at different glucose concentrations (50 to 500 μM), inset shows the corresponding calibration plots.

CV experiments were also conducted with the different amounts of glucose (from 0.1 mM to 0.7 mM) to investigate the concentration effect on the performance of PdNi@rGO/GCE electrode. The relationship between the glucose concentration and the peak intensity of oxidation was given in Fig. 6(a). A proportional relationship was observed between them up to 0.7 mM. The amperometric measurements of PdNi@rGO/GCE electrode were displayed in Fig. 6(b,c). The results were obtained at 0.50 V potential, in 0.1 M NaOH medium, with an increased concentration of glucose (0.1–1.0 mM, with 0.1 mM successive addition steps and, 50 to 500 μM, with 50 μM successive injections). The modified electrode exhibited a rapid response when glucose was introduced and the current became steady-state within 3–5 seconds. The linear correlation between glucose concentration and the current was displayed as inset images. The respective correlation coefficients (R^2^) were found to be very high at 0.9756 and 0.9962. In addition, the detection limit (LOD) of PdNi@rGO/GCE electrode was 0.15 μM, S/N = 3 (Table S1) that supports the proposed sensor have excellent sensitivity and very low detection limit.

#### Electrochemical interference analysis of PdNi@rGO/GCE

**Figure 7:**
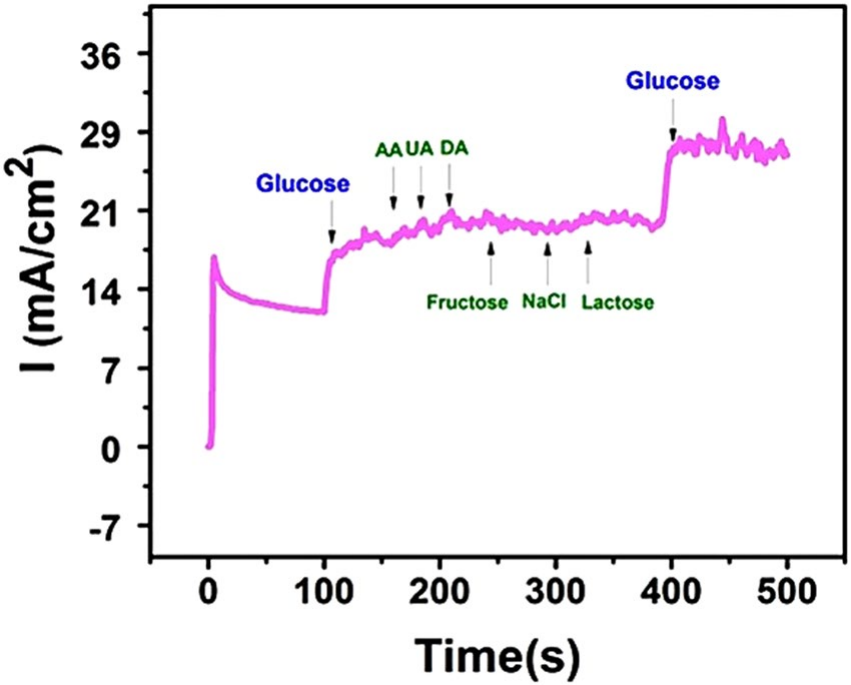
The effect of interferents (with the addition of AA, UA, DA, fructose, NaCl and lactose (0.1 mM for each) respectively) on the amperometric response of PdNi@rGO/GCE. Experiments were conducted at +0.50 V, in alkali medium, and successive addition of 1.0 mM glucose.

In real serum samples, there may be some interferences with some carbohydrates (fructose and lactose) and some biological molecules (e.g. uric acid (UA), dopamine (DA), and ascorbic acid (AA)) which naturally exist in the human blood. Thus, the selectivity of the sensor is a cruel parameter in terms of clinical applicability. Therefore, the interference effect was also examined. For this aim, successive addition of 0.1 mM of AA, DA, fructose, lactose, sodium chloride, and UA was done into 1 mM of glucose. The interference experiment was performed in 0.1 M of NaOH solution at 0.50 V to test the selectivity of PdNi@rGO/GCE. The amperometric i-t curve responses were given in Fig. 7. A significant increase in the amperometric current response was observed with the addition of 1.0 mM glucose. However, the current response did not change slightly with the injection of these potential interferences into the same solution. The responsive signal for additional molecules is very low compared to the glucose signal and these signals can be ignored. The results supported that PdNi@rGO/GCE is highly selective towards the detection of glucose in the coexistence of these interferents.

The applicability of PdNi@rGO/GCE electrode in daily use was investigated with real serum samples which have contain many interference molecules. For these investigations, 10 mL of NaOH (0.1 M) was mixed with 20 µL of human blood serum. PdNi@rGO/GCE electrode was used and amperometric measurements were conducted. The glucose concentrations of each sample were also measured with a commercial glucose monitoring meter designed for using in daily life (GluNEO Lite, IGM-1003A with GDH-FAD test strip enzyme by Infopia Co., Korea) and results were compared in Table 1. Five different samples were used for comparison and it was observed that the proposed sensor showed an excellent agreement with the commercial glucose meter. However, PdNi@rGO/GCE gave slightly higher signal responses than the commercial glucose sensor and the relative standard deviation (N = 5) was found between 1.6 and 5.4%.

**Table 1:** Glucose concentrations in serum samples obtained by PdNi@rGO/GCE electrode and a commercial glucose meter.

Samples	Glucose concentration (mM) (PdNi@rGO/GCE)	Relative standart deviation (RSD) (N = 5)	Glucose concentration (mM) (commercial device)
1	5.50	3.8%	5.30
2	3.50	5.4%	3.32
3	6.00	4.5%	5.74
4	3.10	1.6%	3.05
5	6.75	3.05%	6.55

## Conclusions

In summary, PdNi@rGO nanomaterial was synthesized successfully by a microwave-assisted method for producing a modified GCE that will be utilized in electrochemical glucose detection. A non-enzymatic electrochemical biosensor, PdNi@rGO/GCE, was developed and the used for catalyzed conversion of glucose to gluconolactone, successfully. This novel glucose sensor also showed a low detection limit (0.15 μM), wide linear range (0.05–1.1 mM), a high sensitivity (37.5 mA mM^−1^ cm^−2^ at + 0.5 V), and a very good reproducibility (Table S1). Remarkably, modifiying the GCE surface with PdNi@rGO significantly improved the accuracy and selectivity of the glucose measurement. Moreover, glucose detection with developed electrode did not affected by interferences, and highly satifsied results were obtained in serum samples. Based on the excellent electrochemical performance, it can be suggest that PdNi@rGO/GCE is a promising electrode as a non-enzymatic glucose sensor. The long-term stability tests showed that, after eight weeks keeping in dry and cold conditions, PdNi@rGO/GCE modified electrode indicated excellent stability. In the light of these results, PdNi@rGO/GCE is an excellent candidate for fabricating glucose biosensors in clinical and biotechnology fields.

### Ethical Statement

Authors confirm that all experiments were performed in accordance with relevant guidelines and regulations. Authors confirm that all methods were carried out in accordance with relevant guidelines and regulations. Authors confirm that all experimental protocols were approved by Dumlupınar University Scientific Research and Publication Ethics Committee. Authors confirm that informed consent was obtained from all subjects or, if subjects are under 18, from a parent and/or legal guardian.

## Supplementary information

Supplementary Information
