# Supplementary material for: Highly monodisperse Pd-Ni nanoparticles supported on rGO as a rapid, sensitive, reusable and selective enzyme-free glucose sensor
Source: Sci Rep. 2019 Dec 17;9:19228. doi: 10.1038/s41598-019-55746-y (PMC6917712; doi:10.1038/s41598-019-55746-y)
Supplement: Supplementary file 1 — Supplementary Information [file 41598_2019_55746_MOESM1_ESM.docx]

**SUPPORTING INFORMATION**

**Highly monodisperse Pd-Ni nanoparticles supported on rGO as a rapid, sensitive, reusable and selective enzyme-free glucose sensor**

Aysun Şavk^1^, Kemal Cellat^1^, Kubilay Arıkan^1^, Fatih Tezcan^2^, Senem Karahan Gülbay^3^, Servet Kızıldağ^4^, Elif Şahin Işgın^3,*^, Fatih Şen^1,*^

^1^Sen Research Group, Biochemistry Department, Faculty of Arts and Science, Dumlupınar University, Evliya Çelebi Campus, 43100 Kütahya, Turkey.

^2^Mersin University, Science and Letters Faculty, Chemistry Department, 33343 Mersin, Turkey

^3^Department of Chemistry, Faculty of Sciences, Dokuz Eylul University, Buca, İzmir, Turkey

^4^College of Vocational School of Health Services, Dokuz Eylül University School of Medicine, İzmir, Turkey

^*^Corresponding authors: Fatih Şen and Elif Şahin Işgın, email: fatih.sen@dpu.edu.tr, elif.sahin@deu.edu.tr

Tel:+90 274 265 20 31 -37 02 Fax: +90 274 265 20 56

**Materials and Characterization**

Palladium (II) chloride (PdCl_2_), D-(+)-glucose, graphite, sodium hydroxide (NaOH), hydrochloric acid (HCl), sulfuric acid (H_2_SO_4_), ammonium nitrate (HNO_3_), and Nickel(II) acetate tetrahydrate were purchased from Sigma–Aldrich. All the chemicals were analytical grade and used without any further purification.

Graphene Oxide (GO) was prepared by modified Hummers method as reported in our previous work ^1^. 0.5 g of sodium nitrate and 1 g of graphite were mixed, and 20 ml of concentrated sulfuric acid was added under continuous mixing. After one hour, 3 g of KMnO_4_ was slowly added to keep temperature less than 20°C. The solution was mixed for 2 h at 35 °C and diluted to 100 mL. 5 mL of 30% H_2_O_2_ was poured into the mixture to finalize the reaction with KMnO_4_. After washing of the final mixture with HCl and water, GO sheets were filtered and dried. rRGO was prepared as follows: 100 mg GO was loaded in 250 mL round-bottomed flask, and 100 mL water was added. The dispersion was sonicated for 1 h. Hydrazine hydrate (1.00 mL) was added into the solution and refluxed at 100 ^o^C. The reduced GO was obtained as a black solid. The resultant was filtered, washed with water and ethanol. Finally, it was dried under vacuum at 80 ^o^C.

HR-TEM images of PdNi@rGO have been obtained by a JEOL 200 kV TEM instrument. Sample preparation was carried out through the suspension of ~0.5 mg catalyst in 3 ml of ethanol in an ultrasonic bath and then a drop of this solution was put on to a carbon covered 400-mesh copper grid. Specs spectrometer was used for X-ray Photoelectron Spectroscopy (XPS) measurements and the X-ray source was Kα lines of Mg (1253.6 eV, 10 mA). Samples were prepared by depositing the catalyst on Cu double-sided tape (3M Inc.). C 1s line at 284.6 eV was chosen as a reference point and all XPS peaks were fitted using a Gaussian function. Raman spectrum was obtained via Raman microprobe (Renishaw Instruments) with 514 nm laser excitation.

Electrochemical impedance spectroscopy (EIS) with conventional three electrodes technique was performed in a Gamry (Reference 3000) potentiostat/galvanostat with the frequency range of 1 – 10^5^ Hz in the electrolyte of 1.0 mM [Fe(CN)6]^3−/4-^ which contains 0.1 M KCl as supporting electrolyte. A platinum wire used as a counter electrode and Ag/AgCl (3 M KCl) used as a reference electrode under atmospheric conditions. The exposed surface area of the working electrode was 7.065 mm^2^.


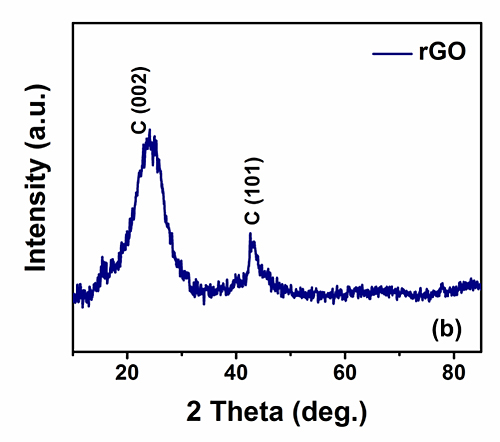


**Figure S1.** XRD pattern of rGO

**Electrochemical properties**

**
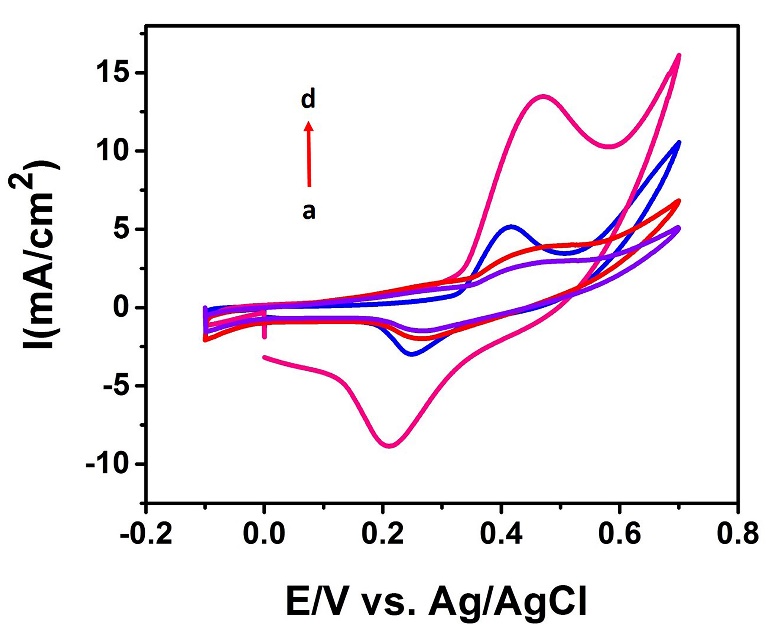
**

**Figure S2.** Cyclic voltammograms obtained for PdNi/GCE (a) without glucose and (b) 0.1 mM glucose; for PdNi@rGO /GCE (c) without glucose and (d) 0.1 mM glucose (at 50 mV/s scan rate in 0.1 M NaOH solution).

**Long Term Stability**

The long-term stability of the sensor was monitored by consecutive amperometric measurements of 0.1 mM glucose for more than eight weeks (Fig. S3). After the 1000 cycle of operation, the sensor has kept 20% of the original signal response, which indicates excellent stability and durability of the Pd-Ni@rGO/GCE electrode for long-term measurements.

| 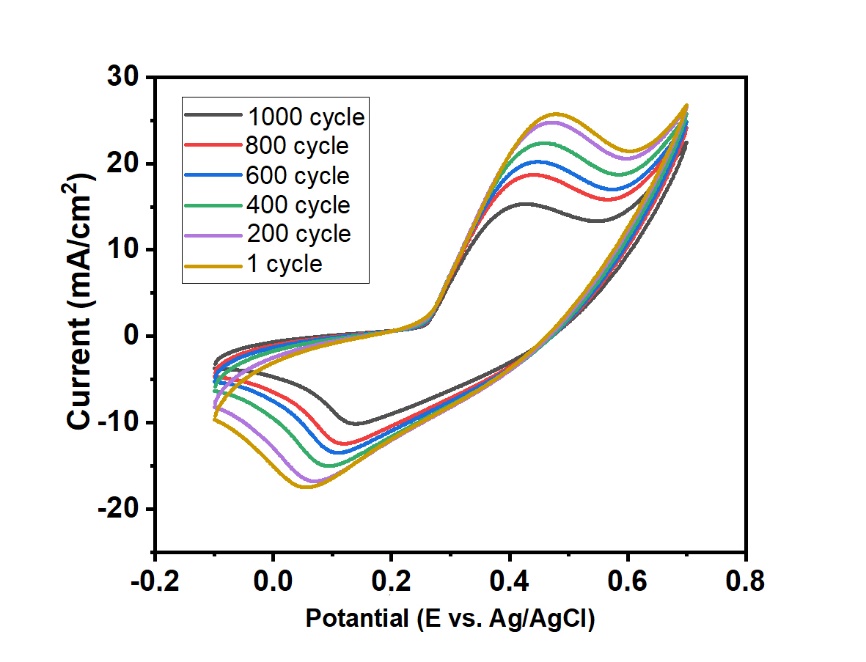  **a)** |
| --- |
| 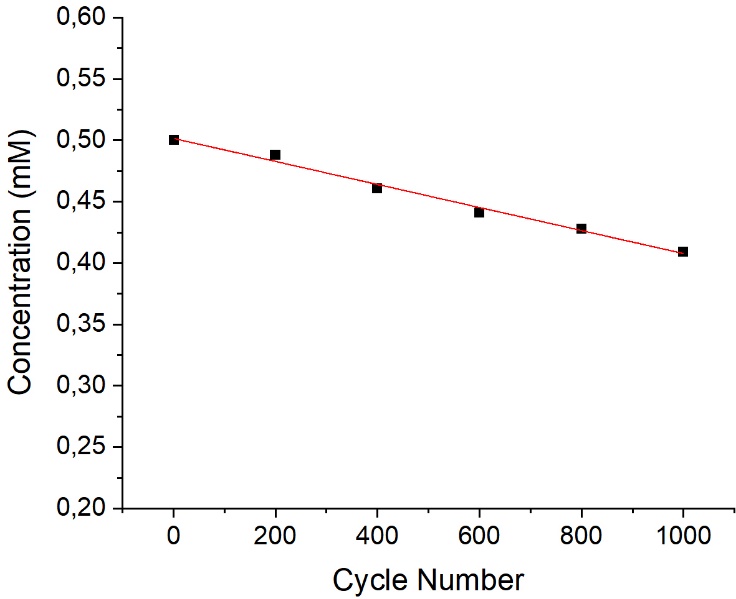  **b)** |

**Fig. S3.** The stability tests of PdNi@rGO/GCE (a) repetitive CV response (b) the change of measured concentration with the cycle number.

**Table S1.** The comparison of PdNi@rGO/GCE with other Ni and Pd based sensors in literature.

| Sensor | Detection limit (μM) | Linear range (μM) | Sensitivity (μA mM^−1^ cm^−2^) | Ref. |
| --- | --- | --- | --- | --- |
| Cu−Pd/GC | 0.32 | 10-9600 | 298 | ^2^ |
| Nano NiO | 0.16 | 1–110 | 55.9 | ^3^ |
| Ni-rGO | 1 | 1–110 | 813 | ^4^ |
| Pd NPs/graphene-CNTs | 1 | Up to 19500 | 110.2 | ^5^ |
| Ni NPs/CNF | 1 | 2 –2500 | 420.4 | ^6^ |
| CuO/NiO/PANI/GCE | 20–2500 | 2 | - | ^7^ |
| Pd NPs/Ni5P4-NF | 0.91 | 2-4650 | 242.5 | ^8^ |
| Pd NPs/GO | - | 200-1000 | - | ^9^ |
| Pd-Pt-graphene/GCE | 5 | 1000-23000 | - | ^10^ |
| Pd NPs on epoxy-silver electrodes | - | 1000-20000 | - | ^10^ |
| GOx–Pd-HCNF/GCE | 30 | 60-6000 | 13000 | ^11^ |
| **PdNi@rGO** | **0.15** | **Up to 1100** | **37.5** | **This work** |

References

1. Aday, B. *et al.* One-pot, efficient and green synthesis of acridinedione derivatives using highly monodisperse platinum nanoparticles supported with reduced graphene oxide. *New J. Chem.* **40**, 748–754 (2016).

2. Jiang, L. C. & Zhang, W. De. A highly sensitive nonenzymatic glucose sensor based on CuO nanoparticles-modified carbon nanotube electrode. *Biosens. Bioelectron.* **25**, 1402–1407 (2010).

3. Mu, Y., Jia, D., He, Y., Miao, Y. & Wu, H.-L. Nano nickel oxide modified non-enzymatic glucose sensors with enhanced sensitivity through an electrochemical process strategy at high potential. *Biosens. Bioelectron.* **26**, 2948–2952 (2011).

4. Wang, Z., Hu, Y., Yang, W., Zhou, M. & Hu, X. Facile one-step microwave-assisted route towards Ni nanospheres/reduced graphene oxide hybrids for non-enzymatic glucose sensing. *Sensors (Basel).* **12**, 4860–9 (2012).

5. Nayak, P., Nair, S. P. & Ramaprabhu, S. Enzyme-less and low-potential sensing of glucose using a glassy carbon electrode modified with palladium nanoparticles deposited on graphene-wrapped carbon nanotubes. *Microchim. Acta* **183**, 1055–1062 (2016).

6. Liu, Y., Teng, H., Hou, H. & You, T. Nonenzymatic glucose sensor based on renewable electrospun Ni nanoparticle-loaded carbon nanofiber paste electrode. *Biosens. Bioelectron.* **24**, 3329–3334 (2009).

7. Ghanbari, K. & Babaei, Z. Fabrication and characterization of non-enzymatic glucose sensor based on ternary NiO/CuO/polyaniline nanocomposite. *Anal. Biochem.* **498**, 37–46 (2016).

8. Wang, M. *et al.* Well-dispersed palladium nanoparticles on nickel- phosphorus nanosheets as efficient three-dimensional platform for superior catalytic glucose electro-oxidation and non-enzymatic sensing. *J. Colloid Interface Sci.* **511**, 355–364 (2018).

9. Wang, Q. *et al.* Well-dispersed palladium nanoparticles on graphene oxide as a non-enzymatic glucose sensor. *RSC Adv.* **2**, 6245 (2012).

10. Zhang, H., Xu, X., Yin, Y., Wu, P. & Cai, C. Nonenzymatic electrochemical detection of glucose based on Pd 1Pt3-graphene nanomaterials. *J. Electroanal. Chem.* **690**, 19–24 (2013).

11. Jia, X. *et al.* Synthesis of palladium/helical carbon nanofiber hybrid nanostructures and their application for hydrogen peroxide and glucose detection. *ACS Appl. Mater. Interfaces* **5**, 12017–12022 (2013).
